# Performance and impact of GeneXpert MTB/RIF® and Loopamp MTBC Detection Kit® assays on tuberculosis case detection in Madagascar

**DOI:** 10.1186/s12879-019-4198-6

**Published:** 2019-06-20

**Authors:** Niaina Rakotosamimanana, Simon Grandjean Lapierre, Vaomalala Raharimanga, Mamy Serge Raherison, Astrid M. Knoblauch, Antso Hasina Raherinandrasana, Andrianantenaina Rakotoson, Julio Rakotonirina, Voahangy Rasolofo

**Affiliations:** 1 Mycobacteria Unit, Institut Pasteur de Madagascar, Antananarivo, Madagascar; 2 Immunopathology axis, Centre de Recherche du Centre Hospitalier de l’Université de Montréal, Montréal, Canada; 3 Epidemiology Unit, Institut Pasteur de Madagascar, Antananarivo, Madagascar; 4 Epidemiology and Public Health Department, Swiss Tropical and Public Health Institute, Basel, Switzerland; 5 Service du Laboratoire des Mycobactéries, Ministry of Public Health, Antananarivo, Madagascar; 6 Centre hospitalier universitaire de soins et de santé publique d’Analakely, Analakely, Antananarivo, Madagascar

**Keywords:** Tuberculosis, Loop-mediated isothermal amplification, GeneXpert, Case detection, Molecular diagnostics

## Abstract

**Background:**

Tuberculosis rapid molecular assays, including GeneXpert MTB/RIF® and Loopamp MTBC Detection Kit®, are highly sensitive and specific. Such performance does not automatically translate in improved disease control and highly depends on their use, local epidemiology and the diagnostic algorithms they’re implemented within. We evaluate the performance of both assays and assess their impact on additional cases notification when implemented within WHO recommended tuberculosis diagnostic algorithms in Madagascar.

**Methods:**

Five hundred forty eight presumptive pulmonary tuberculosis patients were prospectively recruited between November 2013 and December 2014 in Antananarivo, Madagascar, a high TB incidence sub-Saharan African urban setting. Both molecular assays were evaluated as first line or add-on testing following negative smear microscopy. Based on locally defined assay performance characteristics we measure the impact of both assays and WHO-recommended diagnostic algorithms on additional tuberculosis case notifications.

**Results:**

High sensitivity and specificity was confirmed for both GeneXpert MTB/RIF® (86.6% (95% CI 81.1–90.7%) and 97.4% (95% CI 94.9–98.8%)) and Loopamp MTBC Detection Kit® (84.6% (95% CI 78.9–89.0%) and 98.4% (95% CI 96.2–99.4%)). Implementation of GeneXpert MTB/RIF® and Loopamp MTBC Detection Kit® increased tuberculosis diagnostic algorithms sensitivity from 73.6% (95% CI 67.1–79.3%) up to 88.1% (95% CI 82.8–91.9%). This increase was highest when molecular assays were used as add-on testing following negative smear microscopy. As add-on testing, GeneXpert MTB/RIF® and Loopamp MTBC Detection Kit® respectively improved case detection by 23.8 and 21.2% (*p* < 0.05).

**Conclusion:**

Including GeneXpert MTB/RIF® or Loopamp MTBC Detection Kit® molecular assays for TB detection on sputum samples from presumptive TB cases can significantly increase case notification in TB diagnostic centers. The TB case detection rate is further increased when those tests are use as second-line follow-on testing following negative smear microscopy results. A country wide scale-up and digital integration of molecular-based TB diagnosis assays shows promises for TB control in Madagascar.

## Background

In 2017, an estimated 10.0 millions people fell ill with tuberculosis (TB) and approximately 3.6 millions of those went undiagnosed therefore perpetuating the global epidemic [1]. Accurate point-of-care diagnostics are needed to rapidly identify TB cases among presumptive cases, initiate therapy and interrupt person-to-person transmission. Despite lower sensitivity and specificity as compared to novel molecular-based assays, sputum smear microscopy remains the most commonly used TB diagnosis tool globally because of its affordability and potential for implementation at point-of-care. To bypass the inconvenient delays and biosecurity requirements associated with bacterial culture, GeneXpert MTB/RIF® (Cepheid; USA) polymerase chain reaction (PCR) and Loopamp MTBC Detection Kit® (Eiken Chemical Co; Japan) loop-mediated isothermal amplification (LAMP)-based molecular assays were developed, validated on direct sputum samples and endorsed by the World Health Organization (WHO) respectively in 2013 and 2016 [2, 3]. Those assays can either be used as first-line testing in place of sputum smear microscopy or as a follow-on test in adults with symptoms of pulmonary TB but testing negative on smear microscopy [4, 5]. Assays impact on case notifications can vary with context-specific factors including, disease prevalence, population age distribution, HIV rates and implemented diagnosis algorithms.

The objective of this study was to evaluate the diagnostic performance of GeneXpert MTB/RIF® and Loopamp MTBC Detection Kit® assays and assess their impact on case detection when implemented as stand-alone assays for first-line testing or as a follow-on test following negative sputum smear microscopy testing.

## Methods

### Study design

The study participants were recruited in the ‘Établissement Universitaire de Soins et de Santé Publique’ of Antananarivo between November 1^st^, 2013 and December 31^st^, 2014. This healthcare center is part of the National Tuberculosis Control Program (NTP) laboratory network and is audited by the NTP as part of its quality assurance program. Patients 15 years and older were considered for inclusion. Eligible patients were those presenting TB symptoms warranting TB testing as per national guidelines, namely cough for more than 3 weeks with or without hemoptysis and one other TB symptom, such as fever, night sweats, or recent weight loss [6]. Upon inclusion, two (2) morning sputum samples were collected for laboratory testing along with basic epidemiological and clinical data including age, gender and clinical symptoms. Previous or ongoing TB treatment or incapacity to produce at least 4 ml of sputum twice served as exclusion criteria. The study was approved by the ethics committee from the Ministry of Public Health in Madagascar (approval number 102-MSANP/CE). Written informed consent was obtained from all included participants.

### *Mycobacterium tuberculosis* detection testing

All samples were tested in parallel using Lowenstein-Jensen (LJ) solid culture, auramine fluorescence smear microscopy, GeneXpert MTB/RIF®, and Loopamp MTBC Detection Kit®. All assays were performed by trained laboratory technicians according to manufacturer’s recommendations and including positive and negative controls where applicable. Results were interpreted blindly with respect to collateral testing. Fluorescence microscopy was performed and quantitatively interpreted on two immediate consecutive morning sputum samples according to laboratory best practice [7, 8]. Loopamp MTBC Detection Kit® commercial LAMP assay was performed on the first sputum sample. In brief, DNA was extracted from 60 μl of sputum during cyclic heating and adsorption steps. Isothermal amplification was then performed using two sets of four primers targeting six distinct *gyrB* gene and insertion sequences regions of *M. tuberculosis* complex genome. Test result interpretation relies on amplified product visualization under UV irradiation [9, 10]. Study samples were tested prospectively without batching to emulate a clinical laboratory workflow. The remaining sputa were stored at 4°C until being transferred twice weekly to the National Reference Laboratory. GeneXpert MTB/RIF® assay was performed on the second sputum sample. Following sputum homogenization using reaction buffer and vortex, PCR targeting *M. tuberculosis rpoB* gene region was performed in a closed cartridge-based system [11]. In case of “error” or “indeterminate” results, the analysis was repeated on remaining sputum sample if available. Culture was performed after sample N-Acetyl-L-Cysteine Sodium Hydroxide decontamination on the 1^st^ and 2^nd^ collected samples. 200 μl of dissolved specimen solution was inoculated on LJ medium and incubated at 37°C for maximum of twelve weeks [7].

### Diagnostic algorithms and country case notification projections

Three algorithms for the detection of pulmonary TB in adults were evaluated and compared. Smear microscopy as stand-alone assay (algorithm 1) was compared to the two WHO recommended alternatives, namely molecular testing as a replacement of smear microscopy (algorithm 2) or as a follow-on test for smear negative presumptive cases (algorithm 3) [4, 5, 11].

### Statistical analysis

Fluorescence microscopy, GeneXpert MTB/RIF® and Loopamp MTBC Detection Kit® respective diagnostic score values were the primary outcomes. LJ media culture was considered as ‘gold standard’ assay for the analysis. “indeterminate” results on repeated GeneXpert MTB/RIF® testing were excluded from the assay performance analysis. Performance characteristics of both molecular assays were assessed differentially in smear-positive and -negative patients. 95% confidence intervals (CI) were calculated around every assay performance value. Two-tailed chi square test was used to assess performance difference between assays using two-way alpha value of 0.05. All statistical analyses were performed using Stata statistical software version 13.1 (StataCorp LP; College Station, Texas).

## Results

### Study population and samples

**Fig. 1:**
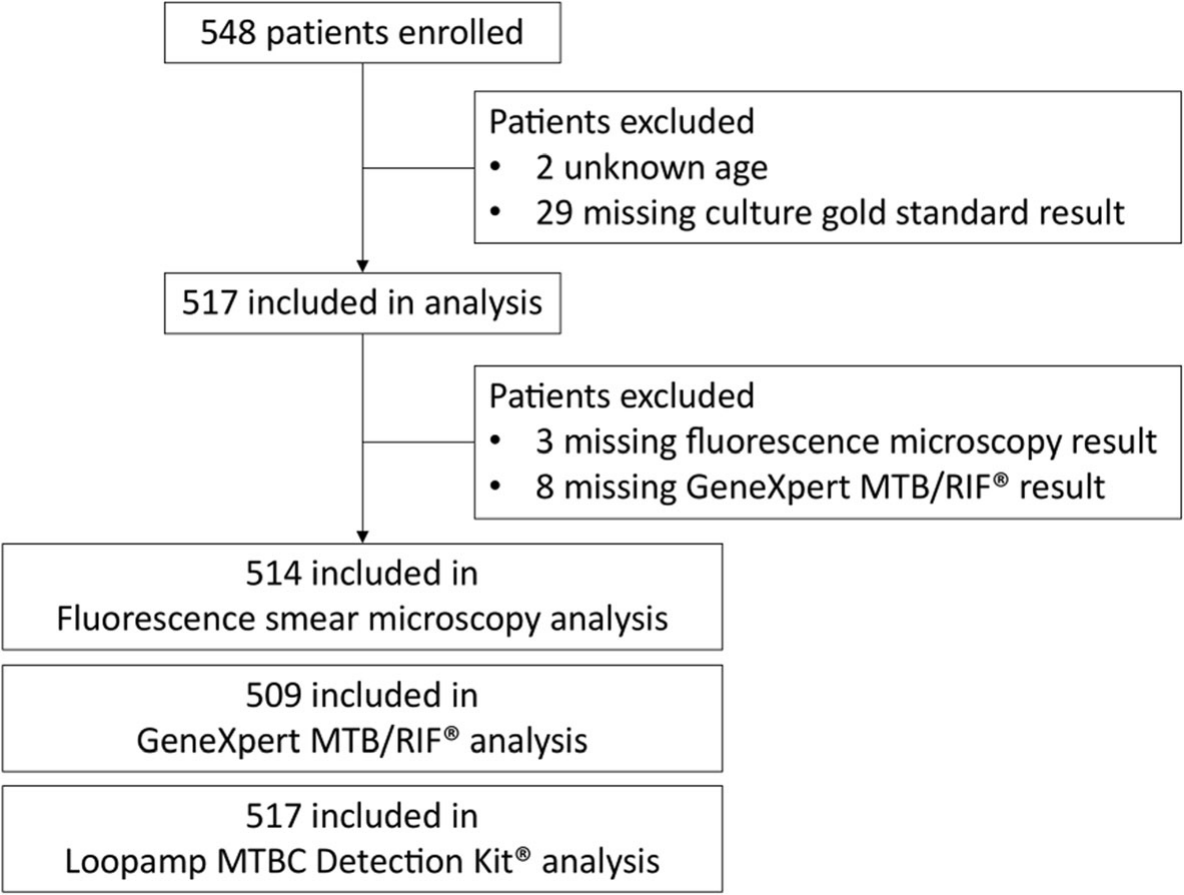
Clinical samples included in the fluorescence smear microscopy, GeneXpert MTB/RIF® and Smear followed by Loopamp MTBC® Detection Kit® performance evaluation. Study enrolment and inclusion process

548 presumptive pulmonary presumptive TB cases were consecutively recruited during the 13-months study period. Sociodemographic and clinical characteristics of the study population are presented in Table 1 – Study population sociodemographic and clinical The median age of the included study population is 40 years old (±16 years old) and the sex ratio is 1.5 (M/F). Chronic cough (more than 3 weeks) was the main clinical symptom observed (85%) that is associated with fever in 57% of the patients (Table 1). Patients were excluded from the study because of unknown age (*n*=2, 0.4%) or missing culture result (n=29, 5,3%). Patients were excluded from assay specific sub-analyses because of missing smear microscopy result (*n*=3, 0,5%) and repeated indeterminate, invalid or error GeneXpert MTB/RIF® results (*n*=8, 1,5%) (Fig. 1 – Clinical samples included in the fluorescence smear microscopy, GeneXpert MTB/RIF® and Smear followed by Loopamp MTBC® Detection Kit® performance evaluation). A total of 201 (38.8%) samples were confirmed to be positive for *M. tuberculosis* on LJ culture.

**Table 1:** Study population sociodemographic and clinical characteristics

Age		
Median	40	
SD^a^	16	
Gender (n and %)		
Female	219	40%
Male	329	60%
Clinical Symptoms (n and %)		
Cough	468	85%
Fever	323	59%
Hemoptysia	135	25%
Chest pain	327	60%
Breathlessness	334	61%
Other	57	10%

^a^ *SD* standard deviation

### Assays clinical performance

Using culture as the gold standard assay, the sensitivity of fluorescence smear microscopy, GeneXpert MTB/RIF® and Loopamp MTBC Detection Kit® assays was, respectively, 73.6% (95% CI 67.1-79.3%), 86.6% (95% CI 81.1-90.7%) and 84.6% (95% CI 78.9-89.0%) (Table 2 - Fluorescence smear microscopy, GeneXpert MTB MTB/RIF ® and Loopamp MTBC Detection Kit® performance). Similarly, assays specificity was, respectively, 99.0% (95% CI 97.1-99.8%), 97.4% (95% CI 94.9-98.8%) and 98.4% (95% CI 96.2-99.4%). Both GeneXpert MTB/RIF® (*p*=0.007) and Loopamp MTBC Detection Kit® were found to be significantly more sensitive than microscopy (*p*=0.001). Between those two assays, however, no significant difference in sensitivity and specificity was observed. When restricting the analysis to smear negative samples, the sensitivity and specificity of GeneXpert MTB/RIF® and Loopamp MTBC Detection Kit® assays were, respectively, 54.7% (95% CI 41.4-67.4%) and 97.4% (95% CI 94.9-98.8%), and 54.7% (95% CI 41.4-67.4%) and 98.4% (95% CI 96.2-99.4%). Oppositely, among smear positive isolates, the sensitivity of GeneXpert MTB/RIF® and Loopamp MTBC Detection Kit® assays, respectively, increased to 98.0% (95% CI 93.9-99.6%) and 95.3% (95% CI 90.4-97.9%). No difference in specificity could be assessed in this sub-group since all smear positive samples were subsequently confirmed to be positive for *M. tuberculosis* in culture (Table 1).

**Table 2:** Fluorescence smear microscopy, GeneXpert MTB MTB/RIF® and Loopamp MTBC Detection Kit® performance

Diagnosis assay	TP	TN	FP	FN	Sensitivity	Specificity	PPV	NPV
	n	n	n	n	% (95% CI)	% (95% CI)	% (95% CI)	% (95% CI)
Fluorescence smear microscopy								
Total (*n* = 514)	148	310	3	53	73.6 (67.1–79.3)	99.0 (97.1–99.8)	98.0 (94.1–99.6)	85.4 (81.4–88.7)
GeneXpert MTB/RIF®								
Smear negative (*n* = 361)	29	300	8	24	54.7 (41.4–67.4)	97.4 (94.9–98.8)	78.4 (62.6–88.9)	92.6 (89.2–95.0)
Smear positive (*n* = 148)	145	0	0	3	98.0 (93.9–99.6)	N/A	100.0 (96.9–100.0)	N/A
Total (*n* = 509)	174	300	8	27	86.6 (81.1–90.7)	97.4 (94.9–98.8)	95.6 (91.4–97.9)	91.7 (88.2–94.3)
Loopamp MTBC Detection Kit®								
Smear negative (*n* = 369)	29	311	5	24	54.7 (41.4–67.4)	98.4 (96.2–99.4)	85.3 (69.4–94.0)	92.8 (89.5–95.2)
Smear positive (*n* = 148)	141	0	0	7	95.3 (90.4–97.9)	N/A	100.0 (96.8–100.0)	N/A
Total (*n* = 517)	170	311	5	31	84.6 (78.9–89.0)	98.4 (96.2–99.4)	97.1 (93.3–99.0)	90.9 (87.4–93.6)

*CI* confidence interval, *FN* false negative, *FP* false positive, *N/A* not applicable, *MTB(C) Mycobacterium tuberculosis* complex, *NPV* negative predictive value, *PPV* positive predictive value, *RIF* rifampicin, *TN* true negative, *TP* true positive

### Programmatic impact of diagnosis algorithms

Madagascar’s current standard of care (algorithm 1), which relies on sputum smear microscopy, diagnosed 151 new cases of pulmonary TB among the 517 included presumptive cases. Implementing a two-step algorithm in which negative smear microscopy testing is systematically followed by molecular testing was found to increase sensitivity and decrease specificity. With GeneXpert MTB/RIF®, sensitivity increased from 73.6% (95% CI 67.1-79.3%) to 88.1% (95% CI 82.8-91.9%; *p*<0.001) and specificity decreased from 99.0% (95% CI 97.1-99.8%) to 96.7% (95% CI 94.0-98.3%; *p*=0.040). With Loopamp MTBC Detection Kit®, sensitivity increased from 73.6% (95% CI 67.1-79.3%) to 88.1% (95% CI 82.8-91.9%; *p*<0.001) and specificity decreased from 99.0% (95% CI 97.1-99.8%) to 97.5% (95% CI 95.0-98.8%, *p*=0.130). Among the 517 included presumptive TB cases, the GeneXpert MTB/RIF® testing-based algorithm would have yielded 8 potential false-positive cases whereas Loopamp MTBC Detection Kit® would have diagnosed 5 such cases, a difference which was not found to be statistically significant (*p*=0.300) (Table 3 - Clinical performance and programmatic impact of three diagnosis algorithms).

**Table 3:** Clinical performance and programmatic impact of three diagnosis algorithms

Algorithm	TP	TN	FP	FN	Sensitivity	Specificity	PPV	NPV	Molecular testing	Additional case notification	Total case notification	LR+	LR-
	n	n	n	n	% (95% CI)	% (95% CI)	% (95% CI)	% (95% CI)	n (%)	n	n		
Algorithm 1													
Smear (n = 514)	148	310	3	53	73.6 (67.1–79.3)	99.0 (97.1–99.8)	98.0 (94.1–99.6)	85.4 (81.4–88.7)	0 (0)	N/A	151	76.8	0.3
Algorithm 2													
GeneXpert MTB/RIF® (*n* = 509)	174	300	8	27	86.6 (81.1–90.7)	97.4 (94.9–98.8)	95.6 (91.4–97.9)	91.7 (88.2–94.3)	509 (100.0)	31	182	33.3	0.1
Loopamp MTBC Detection Kit® (*n* = 517)	170	311	5	31	84.6 (78.9–89.0)	98.4 (96.2–99.4)	97.1 (93.3–99.0)	90.9 (87.4–93.6)	517 (100.0)	24	175	53.5	0.2
Algorithm 3													
Smear - followed by GeneXpert MTB/RIF® (*n* = 506)	177	295	10	24	88.1 (82.8–91.9)	96.7 (94.0–98.3)	94.7 (90.3–97.2)	92.5 (89.0–94.9)	358 (70.1)	36	187	26.9	0.1
Smear - followed by Loopamp MTBC Detection Kit® (*n* = 514)	177	307	6	24	88.1 (82.8–91.9)	97.5 (95.0–98.8)	96.7 (92.9–98.7)	92.8 (89.4–95.1)	366 (71.2)	32	183	45.9	0.1

### Additional TB case detection

**Fig. 2:**
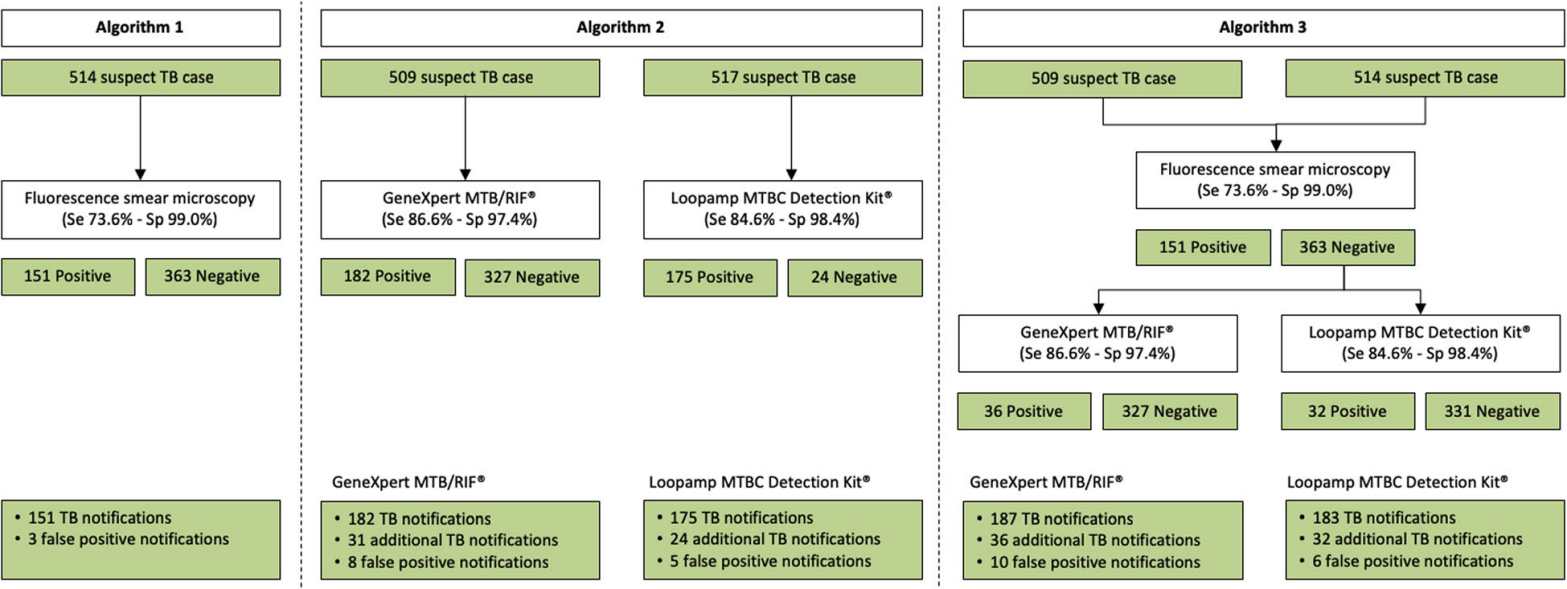
Fluorescence smear microscopy, GeneXpert MTB MTB/RIF® and Loopamp MTBC Detection Kit® based diagnosis algorithms. Analytical performance of three diagnosis algorithms and impact on case notification at study clinic

**Fig. 3:**
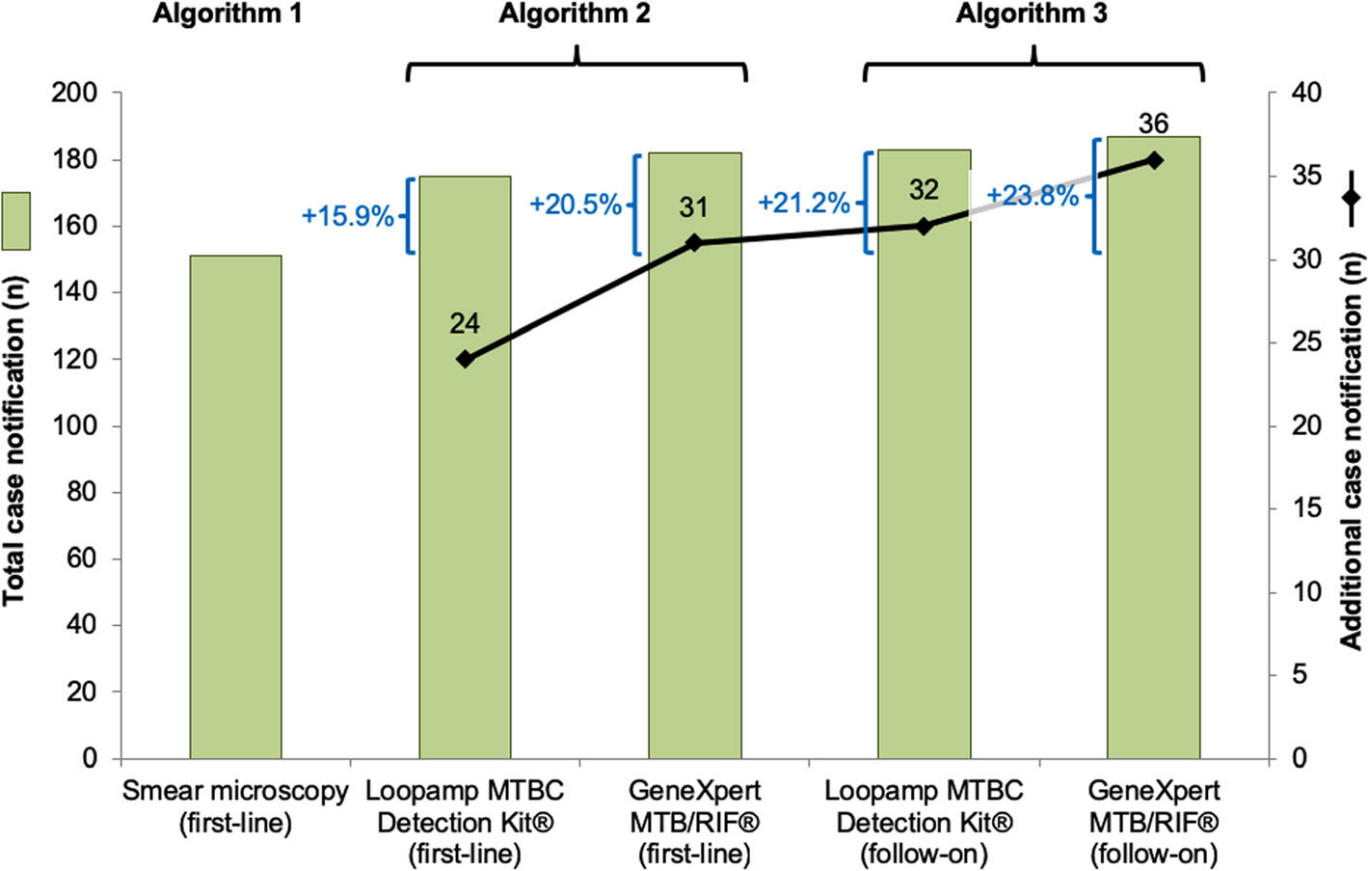
Total and additional pulmonary tuberculosis case notification associated with GeneXpert MTB MTB/RIF® and Loopamp MTBC Detection Kit®. Additional pulmonary tuberculosis notifications associated with evaluated diagnosis algorithms in Établissement Universitaire de Soins et de Santé Publique

Implementing GeneXpert MTB/RIF® or Loopamp MTBC Detection Kit® as first-line assays would have led to total and additional case notifications of 182 and 31 (+20.5%) or 175 and 24 (+15.9%), respectively (Figs. 2 and 3). Using GeneXpert MTB/RIF® or Loopamp MTBC Detection Kit® as follow-on tests following negative smear microscopy would have led to detection of 187 and 36 (+23.8%), and 183 and 32 (+21.2%) total and additional cases, respectively (Fig. 3). The number of additional cases detected is higher when these tests were used as follow-on test following negative smear microscopy compared to its use in the first-line (3.2% and 5.3% respectively for GeneXpert MTB/RIF® and Loopamp MTBC Detection Kit®). This difference between algorithm 2 and 3 was significant for Loopamp MTBC Detection Kit® (*p* = 0.03).

## Discussion

The commercial release and WHO endorsement of PCR and LAMP-based TB nucleic acid detection systems allowed delocalization of such technologies to point-of-care laboratories without previous molecular biology expertise. The capacity of those technologies to rapidly and accurately identify TB directly from clinical samples supported their integration as first-line or follow-on assays for smear-negative presumptive TB [4, 11]. In this study, we consecutively enrolled 548 pulmonary TB suspects, in a clinical and laboratory setting which is representative of the peripheral laboratories where those platforms would eventually be implemented. We evaluated assays performance using 2 sputum cultures as gold standard which represents another strength of this study. Including GeneXpert MTB/RIF® or Loopamp MTBC Detection Kit® molecular assays in the algorithms for TB detection can significantly increase the case notifications in peripheral TB diagnostic centers. This detection rate is further increased when those tests are use as second-line follow-on testing after negative smear microscopy results. The compared diagnosis algorithms did not include differential TB testing approaches for specific populations such as children and patients living with HIV for which the increased sensitivity of molecular diagnosis platforms is desirable. This represents another limitation of this study. HIV incidence is low in Madagascar and the TB-HIV co-infection prevalence recorded by the National TB program in the same study center at the same time of this study was less than 1% [12]. Hence, the presented results still have high internal validity. Further evaluation of additional case notification and costs associated with the use of molecular diagnostic platforms for children should be performed in Madagascar. The WHO’s End TB Strategy calls for universal access to drug susceptibility testing for TB patients [13]. The ability of GeneXpert MTB/RIF® to simultaneously test for both the presence of TB and rifampin resistance represents an important comparative advantage of this platform over Loopamp MTBC Detection Kit® in high MDR-TB prevalence settings or sub-populations [3, 14]. The impact of the difference between simultaneous drug susceptibility testing at point-of care with GeneXpert MTB/RIF® or sequential testing in reference laboratory following Loopamp MTBC Detection Kit® was not assessed in this study.

Delocalizing novel diagnostic assays to peripheral laboratories and TB clinics may negatively impact assays’ performance, hence the necessity for appropriate training during the implementation period and continued quality assurance. GeneXpert MTB/RIF® was confirmed to be more sensitive and specific than fluorescence smear microscopy with sensitivity and specificity reaching 86.6% (95% CI 81.1-90.7%) and 97.4% (95% CI 94.9-98.8%), respectively. These results agree with pooled median sensitivity of 88.0% (95% CI 83.0-92.0%) and specificity of 98.0% (95% CI 97.0-99.0%) reported in a Cochrane Review of fifteen studies assessing the performance of GeneXpert MTB/RIF® on sputum samples [3]. Similarly, with a sensitivity of 84.6% (95% CI 78.9-89.0%) and a specificity of 98.4% (95% CI 96.2-99.4%), Loopamp MTBC Detection Kit® performance was found comparable to reports observed from the literature [2]. For both molecular assays, this agreement between locally assessed performance and reported literature data was maintained when analyzing the smear-positive and smear-negative sub-groups of isolates. In this study, the sensitivity of smear microscopy was found to be higher than usually reported. This could be explained by the fact that analyses were performed in a national reference center by highly trained and experienced personnel in a diagnostic study setting. Unfortunately, this could not be compared to performance in other Malagasy laboratory settings because such data were not available. Nevertheless the measured added value of both evaluated molecular assays could be even higher in other settings. This increased sensitivity of molecular-based diagnosis algorithm is of particular interest for the global community where 3.6 million TB cases still go missing [1, 14].

Among assessed diagnosis algorithms, implementation of GeneXpert MTB/RIF® as follow-on test, yielded the most additional cases with 23.8% additional cases per year. As our data shows, it needs to be emphasized that maintaining smear microscopy as a first-line screening assay has added value on case detection even when implementing new highly sensitive molecular assays. Given WHO’s estimate that only 52% of TB cases are diagnosed in Madagascar, this represents a significant increase [14]. Scaling up molecular diagnostics technologies in Madagascar’s 219 first-line TB diagnostics center certainly represents a challenge and innovative sample transportation systems, continued training and centralized proficiency testing programs would need to accompany a potential diagnosis algorithm change. This study also emphasizes the fact that increased diagnostic assay performance alone cannot be relied upon to find the 48% missing cases. Other public health approaches such as active case finding need to be considered to ensure every presumptive TB case has access to quality diagnostics [15].

Together with diagnostic assays’ analytical performance, operational characteristics and costs are factors which NTP need to consider when designing and implementing diagnosis algorithms. Both molecular assays were found to be easy to implement in delocalized centers without extensive laboratory expertise or previous molecular testing experience. Laboratory results were lost for 11 (2%) samples resulting in partial or complete exclusion of these samples from the analysis. This loss of information occurred between first-line TB clinics and the National Reference Laboratory and is thus unlikely to affect linkage to care and clinical management at local level. Nevertheless, this underlines the importance of appropriate networking of TB laboratory facilities and digitalization of results to facilitate surveillance and diagnosis quality assurance [16]. This study was not designed to assess the cost-effectiveness of molecular diagnosis platforms which were reported to have unit weighted costs of $14.93 USD (GeneXpert MTB/RIF®) and between $13.78 and $16.22 USD (Loopamp MTBC Detection Kit®)) [4, 17, 18]. It can be hypothesized that increased case notification together with rapid treatment initiation could lead to transmission and incidence reduction on the long term.

## Conclusion

Whether as a first-line assay or as follow-on testing for smear-negative TB suspects, GeneXpert MTB/RIF® and Loopamp MTBC Detection Kit® were highly sensitive and specific for the diagnosis of pulmonary TB in Madagascar and proved to significantly increase case detection. The TB case detection rate is further increased when those tests are use as second-line testing following negative smear microscopy results. These platforms are already having a positive impact in Madagascar’s district reference hospitals and now need to be implemented in more remote first-line TB clinics. Strong laboratory network infrastructures including reliable transportation systems, robust proficiency testing and digital results data management will facilitate this transition.

## Data Availability

The dataset supporting of this article is available from the corresponding author upon request.

## Abbreviations

CI: Confidence interval
FIND: Foundation for innovative and new diagnostics
IPM: Institut Pasteur de Madagascar
LAMP: Loop-mediated isothermal amplification
LJ: Lowenstein-Jensen
NTP: National Tuberculosis Control Program
PCR: Polymerase chain reaction
TB: Tuberculosis
WHO: World Health Organization
